# Genomic insights into the diversity, antimicrobial resistance and zoonotic potential of Campylobacter fetus across diverse hosts and geographies

**DOI:** 10.1099/mgen.0.001446

**Published:** 2025-07-10

**Authors:** Ellis Kobina Paintsil, Cynthia Kyerewaa Adu-Asiamah, Kennedy Gyau Boahen, Charity Wiafe Akenten, Alexander Kwarteng, Stefan Berg, Kwasi Obiri-Danso, Jürgen May, Denise Dekker, Linda Aurelia Ofori

**Affiliations:** 1 Roger Williams Institute of Liver Studies, School of Immunology and Microbial Sciences, Faculty of Life Sciences and Medicine, King’s College London, London, UK; 2 Department of Implementation Research, One Health Bacteriology Group, Bernhard Nocht Institute for Tropical Medicine (BNITM), Bernhard-Nocht-Str. 74, 20359 Hamburg, Germany; 3 Kumasi Centre for Collaborative Research in Tropical Medicine (KCCR), South-End, Asuogya Road, 039-5028 Kumasi, Ghana; 4 Department of Clinical Microbiology, Kwame Nkrumah University of Science and Technology, Kumasi, Ghana; 5 Department of Biochemistry and Biotechnology, Kwame Nkrumah University of Science and Technology, Kumasi, Ghana; 6 Department of Theoretical and Applied Biology, Kwame Nkrumah University of Science and Technology, Kumasi, Ghana; 7 German Centre for Infection Research (DZIF), Partner Site Hamburg-Lübeck-Borstel-Riems, 20359 Hamburg, Germany; 8 Tropical Medicine II, University Medical Center Hamburg-Eppendorf (UKE), 20251 Hamburg, Germany; 9 Bernhard Nocht Institute for Tropical Medicine (BNITM), Bernhard-Nocht-Str. 74, 20359 Hamburg, Germany

**Keywords:** antimicrobial resistance (AMR), *Campylobacter fetus*, comparative genomics, horizontal gene transfer (HGT), zoonotic transmission

## Abstract

**Introduction.** *Campylobacter fetus* causes reproductive diseases in livestock and can lead to zoonotic infections such as bacteraemia, particularly in immunocompromised individuals. Despite its significance, its genomic characteristics remain poorly understood. This study analysed 114 publicly available *C. fetus* genomes to provide global insights into genetic diversity, antimicrobial resistance (AMR) and zoonotic risk.

**Results.** A total of 32 distinct sequence types (STs) were identified across 111 of the 114 *C*. *fetus* genomes, spanning 6 continents and diverse hosts (cattle, humans, sheep and reptiles). The majority of strains from cattle (75.6%, *n*/*N*=34/45) were assigned to ST-4, which was the most prevalent overall (*n*=45), while human-associated genomes exhibited the highest diversity with 16 STs. *C. fetus* subsp. *venerealis* (Cfv) and its biovar *intermedius* (Cfvi) genomes clustered closely, forming distinct branches at the biovar level; however, six Cfv genomes were located within Cfvi clades, suggesting a shared ancestry. *C. fetus* subsp. *testudinum* (Cft), primarily isolated from humans (60.0%, *n*/*N*=18/30), exhibited a more diverse genetic profile, with 20 STs. Cfv from North America and Cfvi from South America formed distinct geographic clusters, while * C. fetus* subsp. *fetus* genomes showed no clear geographic patterns, indicating global spread. Pangenomic analysis revealed substantial variation in gene presence/absence in Cft. Five AMR genes were detected, with *tet(O*) (*n*=3) being the most common. A total of 220 plasmid contigs were identified across 47 genomes, predominantly in Cfvi (66.8%, *n*/*N*=147/220) and Cfv (29.1%, *n*/*N*=64/220). Horizontal gene transfer analysis identified 140 genomic islands across 41 genomes, and virulence factor analysis revealed *cheY* as the sole conserved virulence gene across 35 genomes.

**Conclusion.** These findings provide critical insights into the genomic diversity, zoonotic potential and global distribution of *C. fetus*, emphasizing the need for integrated genomic and epidemiological strategies to assess its impact on human and animal health.

**Impact Statement**

*Campylobacter fetus* is a globally significant zoonotic and veterinary pathogen, yet its genomic diversity, antimicrobial resistance (AMR) mechanisms and evolutionary dynamics remain poorly understood. This study provides a comprehensive genomic analysis of *C. fetus*, utilizing 114 publicly available genomes from diverse hosts across 6 continents. Our findings challenge current assumptions about AMR evolution in *C. fetus*, revealing horizontal gene transfer as a key driver of resistance. While AMR genes were identified, their limited presence – combined with the predominance of predicted plasmid contigs in *C. fetus* subsp. *venerealis* and *C. fetus* subsp. *venerealis* biovar *intermedius* – suggests that plasmids may play a significant role in the adaptation or pathogenicity of *C. fetus*. These insights highlight the urgent need for global genomic surveillance to track AMR emergence, improve diagnostics and inform One Health strategies for mitigating *C. fetus* infections in both humans and animals.

## Data Summary

The authors confirm that all supporting data have been provided within the article or through supplementary data files.

## Introduction

*Campylobacter fetus* is a bacterial species comprising three recognized subspecies: *C. fetus* subsp. *fetus* (Cff), *C. fetus* subsp. *venerealis* (Cfv) and *C. fetus* subsp. *testudinum* (Cft). These subspecies are primarily distinguished by host specificity, ecological niche, pathogenicity and genetic features [1]. Cff has a broad host range, including sheep, cattle and humans, where it causes systemic infections and reproductive disorders, such as ovine abortion and bacteraemia in immunocompromised individuals [2,4]. Cfv is host restricted to cattle, colonizing the reproductive tract and causing bovine genital campylobacteriosis, a major concern in livestock due to its impact on fertility and early embryonic loss ([5,6]. Cft, primarily associated with reptiles, is an emerging zoonotic pathogen occasionally implicated in human infections, especially in individuals with close reptile contact [7,8]. Although traditionally considered a zoonotic pathogen, recent genomic evidence suggests that some *C. fetus* lineages may have originated in humans before adapting to livestock during domestication [9]. The Cfv lineage includes a phenotypic variant, *C. fetus* subsp. *venerealis* biovar *intermedius* (Cfvi), which exhibits distinct biochemical traits but lacks sufficient genetic divergence to warrant classification as a separate subspecies [10]. Differentiation among these subspecies requires multilocus sequence typing (MLST), comparative genomic analysis and host-adaptive molecular markers, as standard biochemical tests alone are often insufficient for accurate classification [10].

Whole-genome sequencing has become a crucial tool for exploring the genetic diversity of *Campylobacter* spp., enabling the identification of sequence types (STs), antimicrobial resistance genes (ARGs), virulence factors and potential zoonotic transmission routes [11]. Previous studies have highlighted the presence of resistance to several antibiotics, including tetracycline, streptomycin and fluoroquinolones, and they have also begun to uncover the role of virulence factors in *C. fetus* pathogenicity [12,15]. However, the full extent of its genomic diversity, the mechanisms underlying antimicrobial resistance (AMR) and its zoonotic potential remain poorly understood, particularly in the context of global surveillance and cross-species transmission. Although *C. fetus* harbours several ARGs [16], comprehensive genomic studies are urgently needed to uncover the mechanisms driving AMR dissemination and the factors contributing to the persistence and spread of resistance genes. The role of mobile genetic elements (MGEs), such as plasmids and genomic islands (GIs), in *C. fetus* is an emerging area of interest, with preliminary findings indicating their potential significance in virulence, immune evasion and AMR [17].

Despite the recent advancements in understanding *C. fetus* [14,18], key questions regarding its genomic diversity, AMR and zoonotic potential largely remain unresolved. Further, more comprehensive insights into the mechanisms driving horizontal gene transfer (HGT), the role of MGEs in AMR dissemination and the genomic features underlying host adaptation and pathogenicity are still lacking [13,17, 19]. This study leverages publicly available *C. fetus* genomes to conduct an in-depth comparative genomic analysis, providing a global perspective on its genetic diversity, AMR patterns and zoonotic risk across diverse hosts and geographies. By addressing these critical gaps, our findings will advance the understanding of *C. fetus* as a significant zoonotic and veterinary pathogen, offering valuable insights for global surveillance, public health strategies and animal disease management.

## Methods

### Strain selection

For a comprehensive genomic analysis of *C. fetus*, genomes were retrieved from the Bacterial and Viral Bioinformatics Resource Center (BV-BRC) server (https://www.bv-brc.org/, last accessed on 7 December 2024). The search was performed using the term ‘*C. fetus*’, and the filter was set to include both complete genomes and whole-genome shotgun sequences. To ensure high-quality data, only genomes flagged as good quality by the BV-BRC database were considered. Additionally, genomes were cross-referenced with NCBI taxonomy data to confirm accurate species identification (https://www.ncbi.nlm.nih.gov/datasets/genome/?taxon=196). A total of 114 *C*. *fetus* genomes, isolated from various hosts (cattle, humans, sheep and reptiles) and geographically distributed across 6 continents, met the inclusion criteria and were retrieved for comparative genomic analysis (Table S1).

### Genome annotation and taxonomic identification

All *C. fetus* genomes were annotated using Prokka v1.14.6 [20], with default parameters. The genome annotations were outputted in GFF3 format for downstream analysis. Prokka was employed to predict genes, rRNA, tRNA and other genomic features, and the resulting files were used for subsequent analyses. Taxonomic classification of the genomes was performed using GTDB-Tk (v2.3.2) to assign objective taxonomic classifications based on the Genome Taxonomy Database (GTDB) [21]. Subspecies assignments of Cff, Cfv and Cfvi were further refined through phylogenetic analysis and the use of secondary data [18].

### *In silico* MLST

*In silico* MLST was performed using the open-source tool MLST (https://github.com/tseemann/mlst), which queries the PubMLST database [22]. The tool identifies STs by aligning the genomic data to loci defined in the PubMLST database, using default parameters for allele and ST determination.

### Phylogenetic analysis

Phylogenetic analysis was performed using the Newick file generated from the Roary output, which was visualized as a circular phylogenetic tree using iTOL v6.5.1 [23]. The tree was annotated with information on genomic features and geographical origin to investigate the evolutionary relationships between the genomes.

### Pangenome analysis

Pangenome analysis was conducted using Roary v3.13.0 [24] with a minimum sequence identity threshold of 90% for blastp. Genes were classified into core (present in ≥99% of genomes), soft-core (95–99%), shell (15–95%) and cloud (0–15%) categories. The gene presence/absence matrix generated by Roary was visualized using the Phandango v1.3.1 web-based visualization tool (www.phandango.net) [25].

### *In silico* analysis of AMR, virulence, plasmids and MGEs

AMR genes and virulence factors were identified using Abricate v1.0.1 (https://github.com/tseemann/abricate) with the ResFinder [26] and VFDB [27] databases for AMR and virulence gene prediction, respectively. Default parameters were used for gene identification, and only hits with ≥90% identity and ≥80% coverage were considered for downstream analysis. Additionally, AMRFinderPlus v4.0 (https://github.com/ncbi/amr/wiki), with database v2024-07-22.1, was used to identify *Campylobacter*-specific point mutations in the assemblies [28]. The genomes identified to be harbouring AMR genes were further visualized using Proksee (https://proksee.ca/) [29]. Putative HGT events were identified using Alien Hunter [30].

GIs were predicted using IslandPath-DIMOB v1.0.0 (https://github.com/brinkmanlab/islandpath), a tool designed based on dinucleotide biases and the presence of mobility genes [31]. The tool was run via the command line, with the default parameters and assembled genome sequences in FASTA format as input. GIs were identified based on their characteristic features, such as the presence of mobile elements and atypical dinucleotide frequencies.

Plasmid contigs were predicted using RFPlasmid v0.0.18 (https://klif.uu.nl/rfplasmid) with a plasmid vote score ≥0.6 [32]. The tool identifies chromosomal and plasmid replication genes using CheckM (https://ecogenomics.github.io/CheckM/) and DIAMOND (https://github.com/bbuchfink /diamond) blast and assesses pentamer frequencies and contig sizes. RFPlasmid shows high sensitivity (up to 99% accuracy) and low error rates (0.002–4.66%) for contigs >3 kb. The model was trained on plasmid and chromosomal sequences from 19 species, including *Campylobacter*, and validated with known chromosomal and plasmid contigs from various bacteria.

## Results

### Genome data

A total of 114 high-quality *C. fetus* genomes were retrieved from the BV-BRC database, including 23 complete genomes and 91 whole-genome shotgun sequences (draft genomes). Only genomes with high completeness (>95%) and low contamination (<5%) based on CheckM estimates were included (Table S1). Excluding one outlier (strain RUG14080), genome sizes ranged from 1.7 to 2.1 Mb, with GC content varying between 32.9 mol% and 34.4 mol%. The outlier genome, strain RUG14080, exhibited a genome size of 1.5 Mb and an unusually high GC content of 47.6 mol%, significantly higher than the other genomes. These genomes were isolated from various hosts, with host data available for 79 genomes: the majority originated from cows (55.7%, *n*=44), followed by humans (38.0%, *n*=30), sheep (5.1%, *n*=4) and reptiles (1.3%, *n*=1). Geographical data were available for 104 genomes, with the following distribution: Europe (30.8%, *n*=32), North America (24.0%, *n*=25), South America (15.4%, *n*=16), Asia (15.4%, *n*=16), Oceania (9.6%, *n*=10) and Africa (4.8%, *n*=5). Further details about these genomes are provided in Table S1.

### Taxonomic identification and geographic/host distribution of MLST types

Taxonomic classification of the 114 *C*. *fetus* genomes was performed using GTDB-Tk (v2.3.2), which accurately classified all Cft genomes (*n*=35) and the remaining genomes as *C. fetus* (Table S2). Subspecies assignments for all *C. fetus* genomes were further refined using secondary data [18] and validated through phylogenetic analysis. However, subspecies identification for three genomes could not be confirmed with the current analysis and available data. Of these, two genomes (SRR5279288_bin.84_CONCOCT _v1.1_MAG and first) were confirmed as *C. fetus* using GTDB-Tk, while the third genome (RUG14080) was identified as a different *Campylobacter* species (Fig. S1).

A total of 32 distinct STs were identified across 111 of the 114 confirmed *C. fetus* genomes analysed, spanning 6 continents and multiple host species (Table S3). The distribution varied among the three subspecies. Although Cft is typically associated with reptiles, it was predominantly isolated from humans (51.4%, *n*/*N*=18/35) and exhibited the highest ST diversity (*n*=20). Overall, ST-4 was the most prevalent, representing 45 genomes, followed by ST-3, observed in 8 genomes. A substantial proportion (90.9%, *n*/*N*=40/44) of Cfv and its biovar *intermedius* (Cfvi) were assigned to ST-4, with 75.6% (*n*/*N*=34/45) originating from cattle. Europe exhibited the highest ST diversity (*n*=18), while South America and Africa each showed the lowest diversity (*n*=1), with only ST-4 detected in both regions. In North America, ST-6 and ST-15 were the second most frequently occurring STs, each accounting for 17.4% (*n*/*N*=4/23) of the genomes. Among genomes with host metadata, those associated with humans displayed the greatest diversity, encompassing 16 distinct STs.

### Phylogenetic and pangenomic analyses

**Fig. 1.**
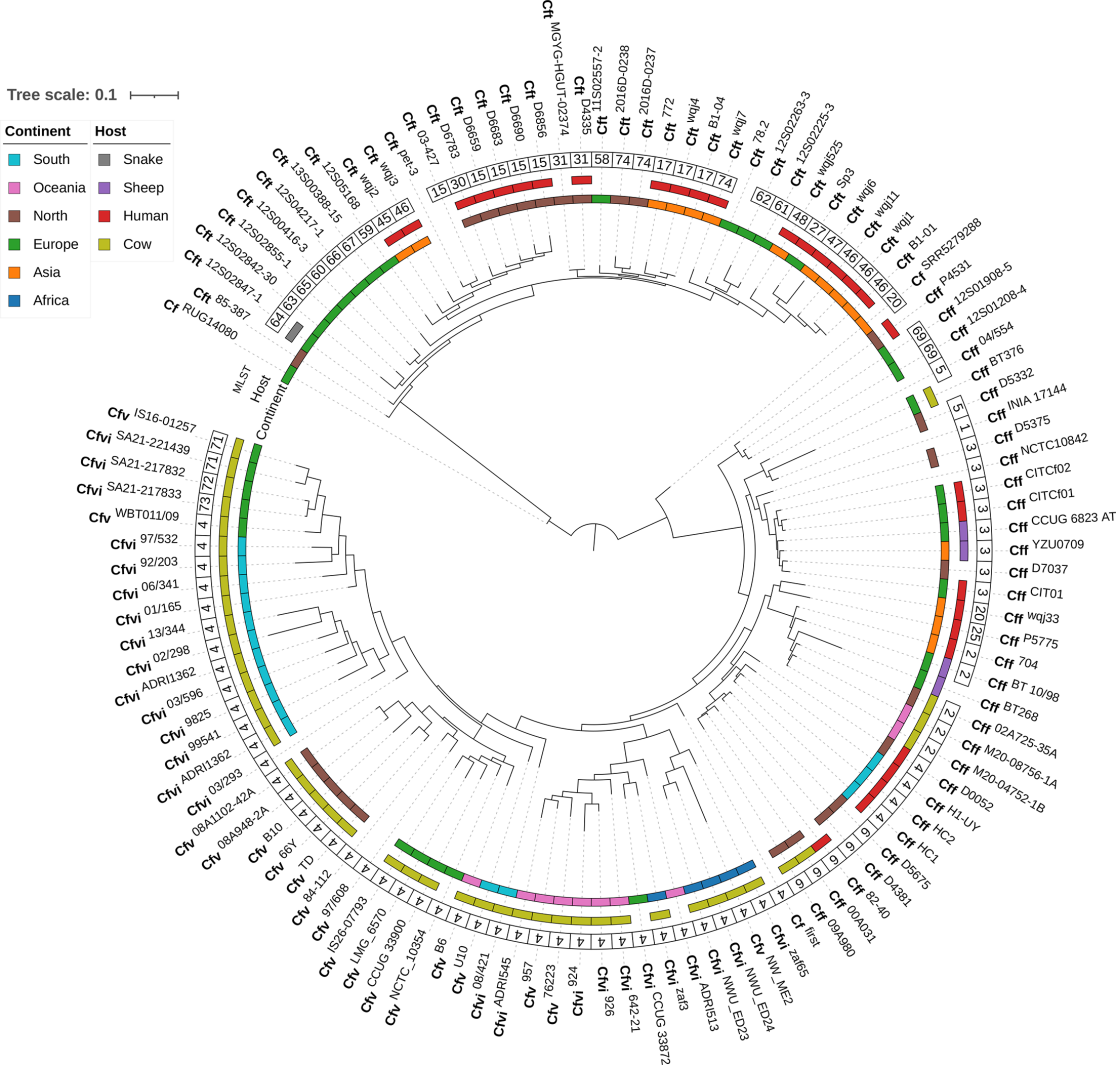
Phylogenetic tree illustrating the evolutionary relationships among the 114 *C*. *fetus* genomes obtained from the BV-BRC database. The tree was constructed using the Newick file generated by Roary, based on core genome SNP analysis, and visualized using the iTOL platform with metadata layers for enhanced interpretability. The inner ring represents the geographic distribution of isolates by continent, with the following colours: Africa (blue), Asia (orange), Europe (green), North America (brown), Oceania (pink) and South America (cyan). The second ring indicates host origins, represented by the following colours: human (red), cow (yellow-green), sheep (purple) and snake (grey), with blank spaces indicating genomes lacking host or geographic metadata. The third ring displays MLST data, and the outer ring depicts taxonomic classification at the species and subspecies levels (Cf, *C. fetus*), with individual strain names displayed in superscript.

The phylogenetic tree, based on core genome SNPs (1,365 genes), illustrates the evolutionary relationships among *C. fetus* subspecies, revealing distinct clustering patterns that highlight genetic diversity and interrelationships (Fig. 1). All Cfv and Cfvi genomes clustered closely despite being isolated from five different continents. While Cfv and Cfvi genomes formed distinct branches at the biovar level, six Cfv genomes were located within Cfvi clades, suggesting a potential shared evolutionary lineage (Fig. S2). In contrast, Cft genomes, which exhibited 20 different STs, clustered together in closely associated clades, entirely distinct from the Cfv, Cfvi and Cff groups. Human-associated genomes were primarily distributed across the Cff and Cft clades, with 60.0% (*n*/*N*=18/30) clustering with Cft and the remaining 40.0% (*n*/*N*=12/30) with Cff. Geographical associations were observed for Cfv and Cfvi genomes. All Cfv genomes from North America clustered on a single branch, while most Cfvi genomes from South America formed a closely related cluster. In contrast, Cff genomes displayed no clear geographical patterns, as branches often contained strains from multiple continents, indicating a more dispersed global distribution.

**Fig. 2.**
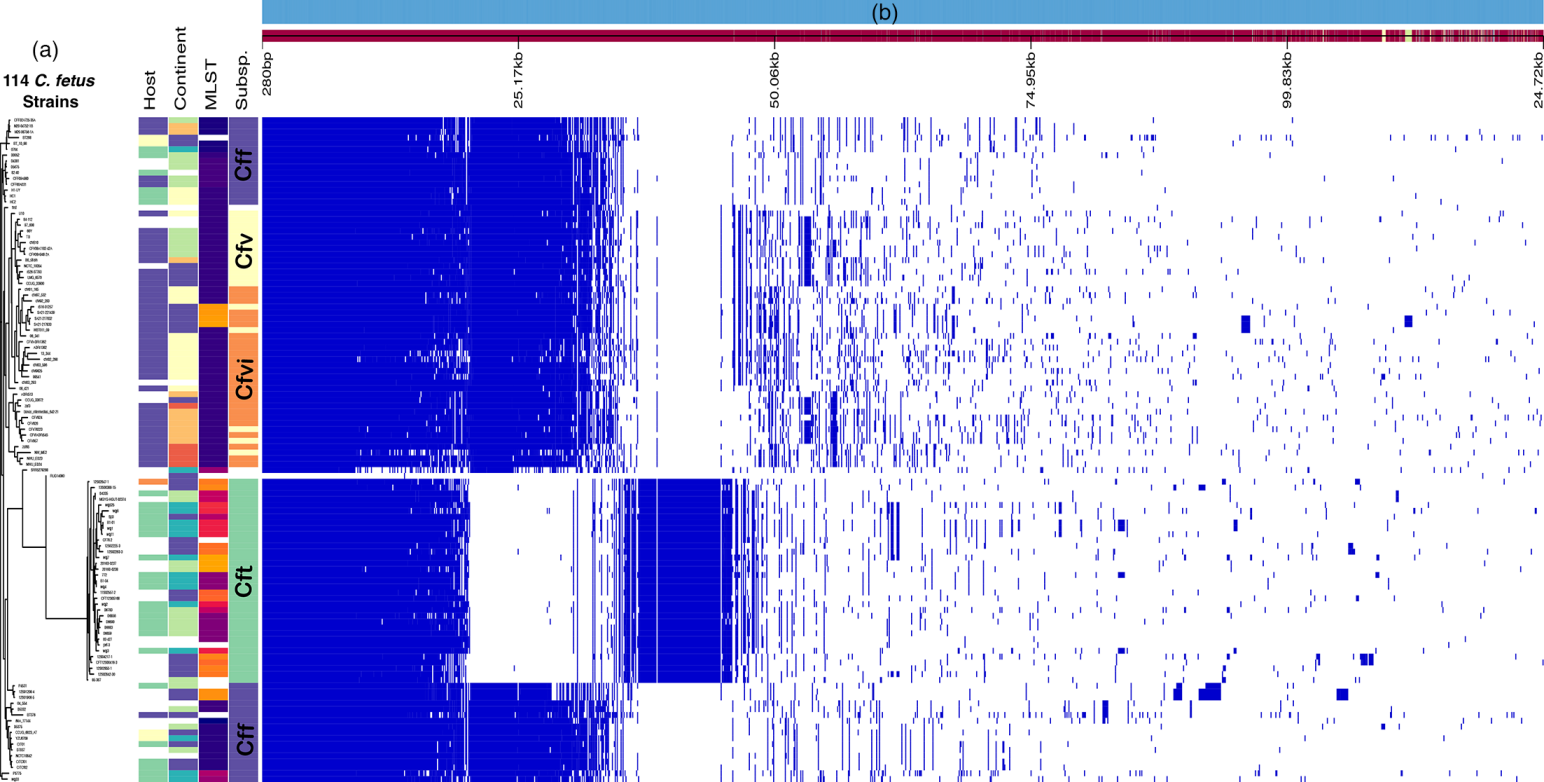
Pangenome analysis of 114 *C*. *fetus* genomes. (**a**) Dendrogram showing the clustering of 114 genomes based on accessory gene distribution, with metadata layers colour coded to indicate host species, geographic origin, MLST and subspecies (Cf, *C. fetus*). (**b**) Roary matrix representing the complete genetic profile of each genome based on the presence/absence of core and accessory genes.

The analysis of 114 *C*. *fetus* genomes identified a total of 9,409 genes, of which 849 were core genes (469 core and 380 soft-core genes) present in at least 95% of the genomes. The remaining 8,560 genes were classified as accessory genes, consisting of 1,866 shell genes (present in 15–95% of strains) and 6,694 cloud genes (present in fewer than 15% of strains). The gene presence/absence matrix revealed distinct patterns among the taxonomic groups. The most pronounced clustering was observed within Cft, where a subset of genes was either unique to Cft or broadly distributed across other subspecies but largely absent in Cft. This distinct gene presence/absence pattern in the pangenome matrix resulted in the segregation of genes exclusive to Cft, as well as those absent in Cft but present in other *C. fetus* subspecies, highlighting its genomic divergence (Fig. 2).

## ARG, virulence factors and MGE

### ARG profiling

ARG analysis across the 114 *C*. *fetus* genomes was conducted using Abricate, revealing the presence of 5 distinct ARGs. The most commonly identified gene was *tet(O*) (*n*=3), detected exclusively in human isolates, which confers resistance to tetracyclines. The distribution of these ARGs was geographically diverse, with genes found in genomes from Asia (*n*=3) and North America (*n*=2) (Table 1). The two North American strains each harboured two distinct AMR genes. A comprehensive summary of the ARGs, their genomic locations and corresponding accession numbers is provided in Table S4. Additionally, using AMRFinderPlus, a point mutation (rpsL_K43R) associated with streptomycin resistance in the *rpsL* gene was detected in strain CFF09A980.

**Table 1.** Distribution and frequency of ARGs in *C. fetus* genomes

Gene	Antibiotic resistance conferred	Host	Region	Frequency
*tet(O*)	Doxycycline, tetracycline, minocycline	Human	Asia, North America	3
*tet(44*)	Doxycycline, tetracycline, minocycline	Cow	North America	1
*ant(6)-Ib*	Streptomycin	Cow	North America	1
*aph(3′)-III*	Amikacin	na	North America	1
*lnu(C*)	Lincomycin	na	Asia	1

na, not available.

### HGT and GIs

**Fig. 3.**
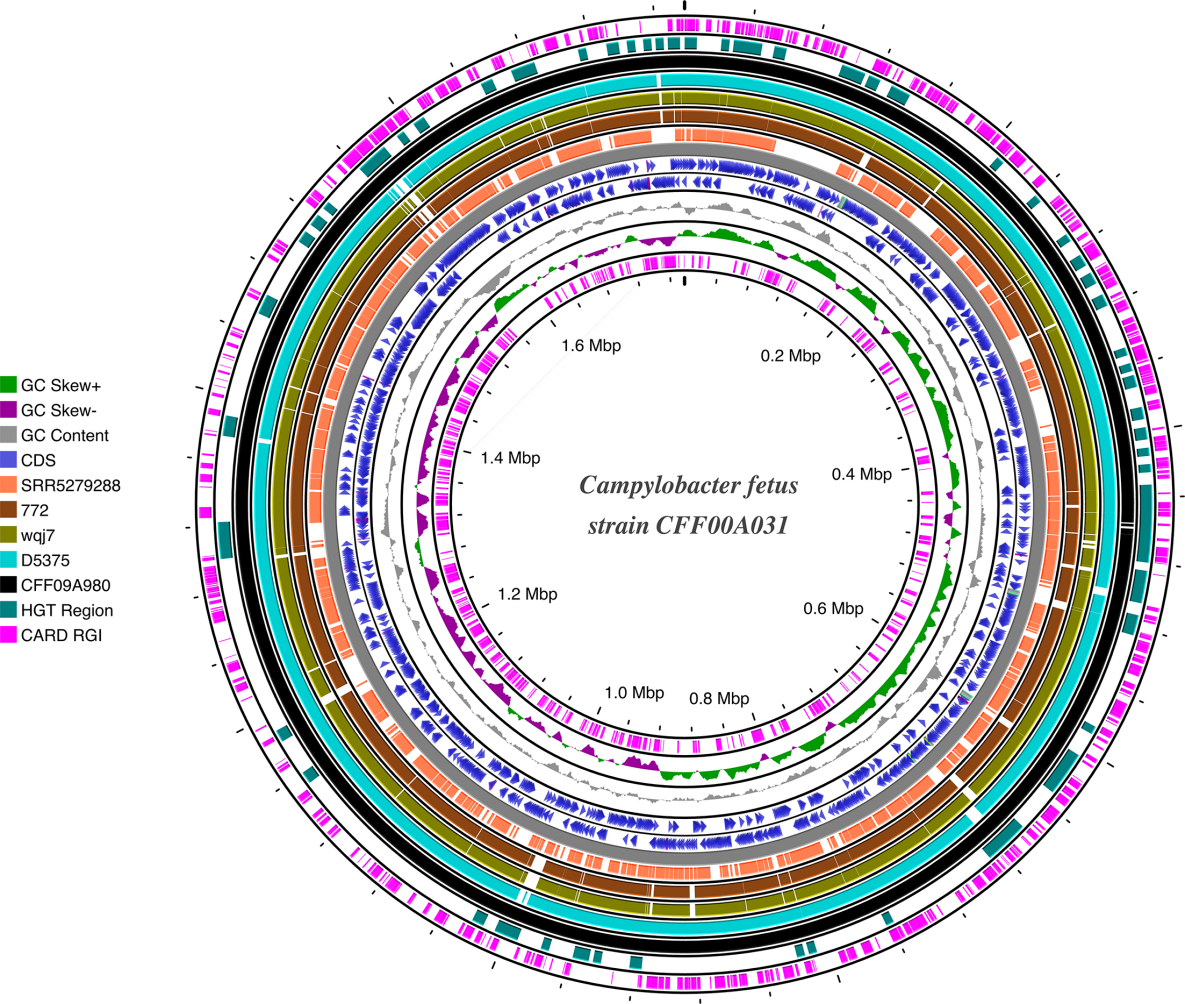
Circular genome representation of the reference genome CFF00A031 compared with five genomes harbouring AMR genes. The circular visualization highlights key genomic features across multiple rings. Starting from the innermost ring: (1) predicted resistance mechanisms (pink), (2) GC skew (purple and green), (3) GC content (grey), (4 and 5) coding sequences (CDS) (two blue rings) and (6) genome backbone (solid grey). Followed by the blastn comparison results of the five AMR-harbouring genomes in the following order: SRR5279288 (coral), 772 (brown), wqj7 (greenish-yellow), D5375 (light blue-green) and CFF09A980 (black). HGT events are indicated in the next ring marked in cyan, with the outermost ring representing the predicted AMR genes highlighted in pink.

An in-depth analysis of the five genomes harbouring AMR genes using Proksee revealed several HGT regions. The CARD RGI [Comprehensive Antibiotic Resistance Database (CARD) Resistance Gene Identifier (RGI)] further elucidated the resistance mechanisms associated with these ARGs, highlighting a diverse array of resistance pathways (Fig. 3). A total of 140 GIs were identified across 41 genomes, emphasizing the widespread presence of HGT elements. The distribution of GIs varied across subspecies: Cff genomes contained the highest proportion (46.3%, *n*=19), while Cft genomes exhibited the lowest number (12.2%, *n*=5) (Table S5).

### Virulence factors

A virulence factor analysis of all 114 *C*. *fetus* genomes identified *cheY*, a key gene in the chemotaxis signalling pathway, as the sole virulence factor present. This gene was found in 35 strains across various hosts and regions (Table S6).

### Plasmid prediction

RFPlasmid analysis predicted a total of 220 plasmid contigs across 47 of the 114 (41.2%) analysed *C. fetus* genomes. The majority of these genomes were Cfvi (51.1%, *n*/*N*=24/47) and Cfv (31.9%, *n*/*N*=15/47), and accordingly, the majority of predicted plasmid contigs were found in Cfvi (66.8%, *n*/*N*=147/220) and Cfv (29.1%, *n*/*N*=64/220). Plasmid contig lengths ranged from 0.99 to 91.4 kb, with an average of nine plasmid marker genes per contig. Most predicted plasmids had a high proportion of plasmid marker genes (median 0.89) and low proportions of chromosomal marker genes (median 0.36), supporting their classification as plasmids. Interestingly, all of the predicted plasmid contigs showed no significant hits against the PlasmidFinder database, with only four (1.8%, *n*/*N*=4/220) contigs showing weak similarity, with their identity percentages ranging from 31.2% to 38.8% (Table S7).

## Discussion

In this study, we analysed 114 publicly available *C. fetus* genomes and observed pronounced geographic and host-associated variation in ST distribution. Europe exhibited the greatest ST diversity, while South America and Africa were dominated by ST-4, primarily among cattle-associated Cfv and Cfvi isolates. These genomes originated from three BioProjects, which may reflect limited regional surveillance or localized outbreaks rather than a true lack of diversity [2,33,35]. In contrast, the broad ST diversity among human-associated Cft, spanning multiple continents, highlights its ecological plasticity and zoonotic potential [36]. These patterns suggest that the global distribution of *C. fetus* is shaped by both host specificity and anthropogenic factors such as livestock trade [37], as well as by the species’ intrinsic capacity for homologous recombination and interspecies transmission [38]. While Cfv and Cfvi exhibited clonal population structures and regional clustering – likely reflecting host restriction and vertical transmission – Cff and Cft displayed broader dispersal patterns and greater ST diversity, consistent with wider host ranges and increased recombination rates [7]. These dynamics resemble the population structure of *Helicobacter pylori*, where regional lineages are shaped by recombination and human migration [39]; however, the zoonotic and environmentally resilient nature of *C. fetus* indicates that its global spread could be more influenced by cross-species transmission and agricultural networks than by human ancestry alone.

Our phylogenomic analysis revealed subspecies-specific clustering patterns that offer insight into the evolutionary and ecological dynamics of *C. fetus*. The tight clustering of Cfv and Cfvi genomes across geographically diverse regions suggests limited genetic exchange and strong host adaptation, likely driven by conserved genomic features linked to immune evasion or metabolic specialization [18,40]. However, the placement of six Cfv genomes within Cfvi clades raises questions regarding the influence of HGT, recombination events or potential biovar misclassification [17,18]. This genomic overlap challenges traditional subspecies boundaries and suggests that current classification frameworks may not fully capture the complexity of genetic relationships between Cfv and Cfvi, potentially due to shared evolutionary origins and ecological adaptations [41,42]. In contrast, Cff genomes were more phylogenetically dispersed, consistent with reduced host specificity and greater environmental resilience [43,44]. Pangenome analysis revealed distinct gene presence/absence profiles within Cft, including unique gene sets absent from other subspecies, further supporting its genomic distinctiveness. The high genomic and ecological plasticity of Cft is also evident in its capacity to colonize diverse hosts, supported by its pronounced gene content variability and ST diversity [45,47]. The distribution of human-associated strains across both Cff and Cft clades highlights their zoonotic potential and suggests independent evolutionary pathways for interspecies transmission [38]. Together, these findings underscore the complex interplay of evolutionary, ecological and anthropogenic forces shaping *C. fetus* populations and emphasize the need for integrative genomic and epidemiological surveillance to refine taxonomy and inform public health strategies.

The ARG profiling across the 114 *C*. *fetus* genomes in this study revealed a relatively limited but geographically diverse presence of ARGs, highlighting the potential for AMR spread within this species. The presence of ARGs in *C. fetus* strains isolated from both human and animal hosts emphasizes their ability to acquire and maintain resistance traits across different host species [16,48]. Given the limited number of strains harbouring these genes, further surveillance of *C. fetus* in diverse geographic regions and host populations is crucial to better understand the dynamics of AMR dissemination and its potential public health impact. Several HGT regions were identified in the analysed *C. fetus* genomes, which provides further insight into the mechanisms underlying ARG acquisition and dissemination [19]. In line with the current findings, an earlier study suggested that *C. fetus* may acquire resistance genes through genetic exchange with other micro-organisms [49]. The widespread distribution of GIs underscores the importance of HGT in shaping the genetic landscape of *C. fetus,* suggesting that species may be more prone to acquiring foreign genetic material, potentially enhancing its adaptability and survival in different ecological niches [50]. Predicted plasmid contigs were identified in nearly half of the *C. fetus* genomes, with a notable predominance in Cfvi and Cfv. This pattern suggests a potential role for plasmids in the adaptation or pathogenicity of venerealis-associated subspecies [13], possibly due to reduced barriers to exogenous DNA uptake in Cfv/Cfvi [51]. The absence of significant matches in the PlasmidFinder database points to a diverse and largely uncharacterized * C. fetus* plasmidome, underscoring the need for expanded reference datasets. Further investigation into the role of plasmid and other MGEs in the evolution of AMR in *C. fetus* is essential to unravel the full scope of resistance mechanisms in this pathogen.

Virulence factor analysis revealed that *C. fetus* strains were only harbouring *cheY*, a key gene involved in the chemotaxis signalling pathway, which plays a crucial role in bacterial motility and host colonization [52]. This gene was present in 35 strains across a range of hosts and geographic regions, suggesting its potential role in facilitating *C. fetus* adaptation to diverse environments and hosts. The widespread distribution of *cheY* in both human and animal-associated strains highlights its importance in the pathogenicity of * C. fetus* and its ability to colonize different host species [53,54]. However, the limited number of virulence factors identified in this study suggests that *C. fetus* may rely on other yet-to-be-identified factors for its pathogenicity, and further studies are warranted to explore the full repertoire of virulence determinants in this species. The presence of virulence factors in *C. fetus* strains from both human and animal hosts also reinforces the zoonotic potential of this pathogen, suggesting that cross-species transmission may be facilitated by the presence of conserved virulence traits [55,56]. Additionally, the identification of a single virulence factor in this study contrasts with the more complex virulence profiles observed in other *Campylobacter* spp., highlighting the unique pathogenic strategies employed by *C. fetus*. Understanding the role of *cheY* and other potential virulence factors in *C. fetus* pathogenicity will be essential for developing targeted interventions to mitigate the impact of this pathogen on both human and animal health.

While this study provides valuable insights into the genomic diversity, plasmid content, AMR and virulence potential of *C. fetus*, several limitations should be acknowledged. First, the relatively small sample size of 114 genomes, although geographically and host-diverse, may not fully capture the species’ genetic variability across different environments or over time. While RFPlasmid offers high sensitivity in plasmid prediction, its accuracy decreases for short contigs and when reference data updates are not performed regularly. Predictions should be regarded as potential plasmids requiring experimental validation. Additionally, reliance on publicly available genomic data introduces potential biases, as these genomes may not represent the entire *C. fetus* population, especially in under-sampled regions or host species. The absence of detailed phenotypic data, such as antimicrobial susceptibility or virulence assays, limits the ability to correlate ARGs and virulence factors with pathogenicity. Finally, while subspecies classification was based on GTDB-Tk, secondary data and phylogenetic analysis, confirmation using more robust methods like Kraken2 would have ensured more precise subspecies identification. Future studies incorporating larger, more diverse datasets, phenotypic data and longitudinal sampling are essential for a comprehensive understanding of *C. fetus* evolution, host adaptation and AMR mechanisms.

## Conclusion

This study provides a comprehensive genomic analysis of *C. fetus*, revealing significant genetic diversity, AMR profiles and zoonotic potential across diverse geographic regions and host species. Our findings highlight the complexity of *C. fetus* subspecies classification, with evidence of HGT and possible subsp. misclassification, suggesting the need for more robust *in silico* methods for subspecies identification. While ARGs were identified, their limited presence, combined with the predominance of predicted plasmid contigs in Cfvi and Cfv, suggests that plasmids could play a significant role in the adaptation or pathogenicity of *C. fetus*. Despite relying on publicly available genomes and the absence of phenotypic data, our findings emphasize the importance of integrating genomic, epidemiological and phenotypic approaches to better understand *C. fetus* evolution, host adaptation and AMR mechanisms, informing strategies to mitigate its impact on human and veterinary health.

## Supplementary material

10.1099/mgen.0.001446 Uncited Supplementary Material 1.

10.1099/mgen.0.001446 Supplementary Material 2.

## Abbreviations

AMR: antimicrobial resistance
ARG: antimicrobial resistance gene
BV-BRC: Bacterial and Viral Bioinformatics Resource Center
Cff: *Campylobacter fetus* subsp. *fetus*
Cft: *Campylobacter fetus* subsp. *testudinum*
Cfv: *Campylobacter fetus* subsp. *venerealis*
Cfvi: *Campylobacter fetus* subsp. *venerealis* biovar *intermedius*
GIs: genomic islands
HGT: horizontal gene transfer
MGE: mobile genetic element
MLST: multilocus sequence typing
ST: sequence type
