# Supplementary material for: Genomic insights into the diversity, antimicrobial resistance and zoonotic potential of Campylobacter fetus across diverse hosts and geographies
Source: Microb Genom. 2025 Jul 10;11(7):001446. doi: 10.1099/mgen.0.001446 (PMC12244366; doi:10.1099/mgen.0.001446)
Supplement: Uncited Supplementary Material 1. [file mgen-11-01446-s001.pdf]

Supplementary Figures

Genomic Insights into the Diversity, Antimicrobial Resistance, and Zoonotic Potential of *Campylobacter fetus* Across Diverse Hosts and Geographies

Ellis Kobina Paintsil[1,2,*], Cynthia Kyerewaa Adu-Asiamah[3], Kennedy Gyau Boahen[4], Charity Wiafe Akenten[3], Alexander Kwarteng[5], Stefan Berg[2], Kwasi Obiri-Danso[6], Jürgen May[7,8,9], Denise Dekker[2], Linda Aurelia Ofori[6]

Authors' information

[1]Roger Williams Institute of Liver Studies, School of Immunology and Microbial Sciences, Faculty of Life Sciences and Medicine, King's College London, UK

[2]Department of Implementation Research, One Health Bacteriology Group, Bernhard Nocht Institute for Tropical Medicine (BNITM), Bernhard-Nocht-Str. 74, 20359 Hamburg, Germany

[3]Kumasi Centre for Collaborative Research in Tropical Medicine (KCCR), South-End, Asuogya Road, 039-5028 Kumasi, Ghana

[4]Department of Clinical Microbiology, Kwame Nkrumah University of Science and Technology, Kumasi, Ghana

[5]Department of Biochemistry and Biotechnology, Kwame Nkrumah University of Science and Technology, Kumasi, Ghana

[6]Department of Theoretical and Applied Biology, Kwame Nkrumah University of Science and Technology, Kumasi, Ghana

[7]Bernhard Nocht Institute for Tropical Medicine (BNITM), Bernhard-Nocht-Str. 74, 20359, Hamburg, Germany

[8]German Centre for Infection Research (DZIF), Partner Site Hamburg-Lübeck-Borstel-Riems, 20359, Hamburg, Germany

[9]Tropical Medicine II, University Medical Center Hamburg-Eppendorf (UKE), 20251, Hamburg, Germany

* Correspondence: ellis.paintsil@kcl.ac.uk

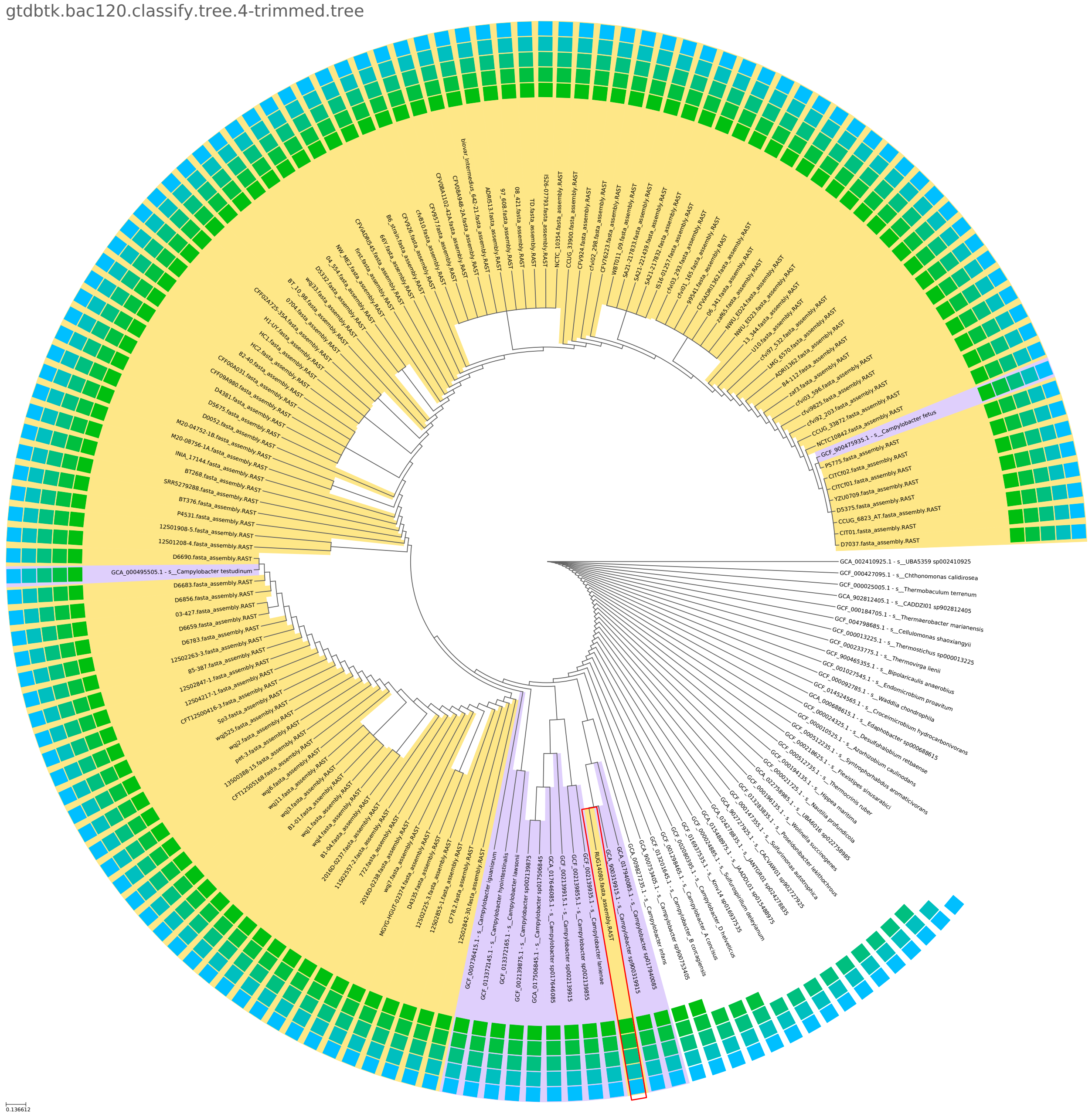

Figure S1: Taxonomic Identification of 114 *C. fetus* Genomes Using KBase, Highlighting Strain RUG14080 (Red) as a Different *Campylobacter* Species

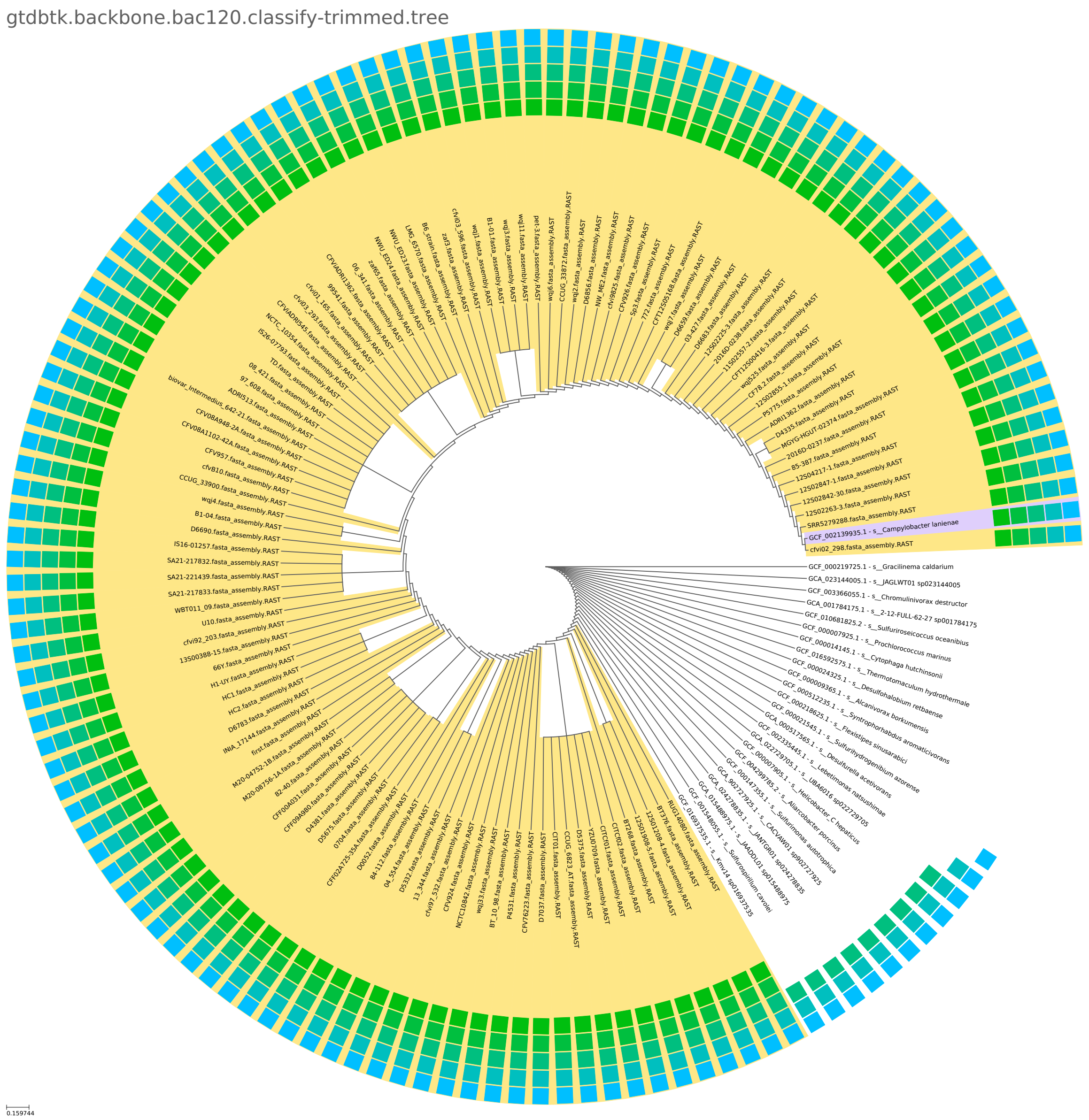

Figure S2: Phylogenetic tree of 114 *C. fetus* genomes generated with GTDB-Tk v2.3.2, with branch lengths indicating evolutionary distance.
